# A20 deficiency causes spontaneous neuroinflammation in mice

**DOI:** 10.1186/1742-2094-11-122

**Published:** 2014-07-16

**Authors:** Renata Padilha Guedes, Eva Csizmadia, Herwig P Moll, Averil Ma, Christiane Ferran, Cleide Gonçalves da Silva

**Affiliations:** 1 Division of Vascular Surgery, Center for Vascular Biology Research and the Transplant Institute, Department of Surgery, Beth Israel Deaconess Medical Center, Harvard Medical School, Boston, MA, USA; 2 Division of Gastroenterology, University of California, San Francisco, CA, USA; 3 Current address: Federal University of Health Sciences of Porto Alegre (UFCSPA), Porto Alegre, RS, Brazil

**Keywords:** TNFAIP3/A20, Neuroinflammation, Reactive gliosis, Inflammatory cytokines, Oxidative stress

## Abstract

**Background:**

A20 (*TNFAIP3*) is a pleiotropic NFκB-dependent gene that terminates NFκB activation in response to inflammatory stimuli. The potent anti-inflammatory properties of A20 are well characterized in several organs. However, little is known about its role in the brain. In this study, we investigated the brain phenotype of A20 heterozygous (HT) and knockout (KO) mice.

**Methods:**

The inflammatory status of A20 wild type (WT), HT and KO brain was determined by immunostaining, quantitative PCR, and Western blot analysis. Cytokines secretion was evaluated by ELISA. Quantitative results were statistically analyzed by ANOVA followed by a *post-hoc* test.

**Results:**

Total loss of A20 caused remarkable reactive microgliosis and astrogliosis, as determined by F4/80 and GFAP immunostaining. Glial activation correlated with significantly higher mRNA and protein levels of the pro-inflammatory molecules TNF, IL-6, and MCP-1 in cerebral cortex and hippocampus of A20 KO, as compared to WT. Basal and TNF/LPS-induced cytokine production was significantly higher in A20 deficient mouse primary astrocytes and in a mouse microglia cell line. Brain endothelium of A20 KO mice demonstrated baseline activation as shown by increased vascular immunostaining for ICAM-1 and VCAM-1, and mRNA levels of E-selectin. In addition, total loss of A20 increased basal brain oxidative/nitrosative stress, as indicated by higher iNOS and NADPH oxidase subunit gp91^phox^ levels, correlating with increased protein nitration, gauged by nitrotyrosine immunostaining. Notably, we also observed lower neurofilaments immunostaining in A20 KO brains, suggesting higher susceptibility to axonal injury. Importantly, A20 HT brains showed an intermediate phenotype, exhibiting considerable, albeit not statistically significant, increase in markers of basal inflammation when compared to WT.

**Conclusions:**

This is the first characterization of spontaneous neuroinflammation caused by total or partial loss of A20, suggesting its key role in maintenance of nervous tissue homeostasis, particularly control of inflammation. Remarkably, mere partial loss of A20 was sufficient to cause chronic, spontaneous low-grade cerebral inflammation, which could sensitize these animals to neurodegenerative diseases. These findings carry strong clinical relevance in that they question implication of identified A20 SNPs that lower A20 expression/function (phenocopying A20 HT mice) in the pathophysiology of neuroinflammatory diseases.

## Background

Neuroinflammation is a common pathogenic culprit of several neurodegenerative diseases including Alzheimer’s Disease (AD) [1], Parkinson’s Disease (PD) [2], multiple sclerosis (MS) [3], stroke [4] and neuropsychiatric diseases such as depression, schizophrenia and autism [5,6]. Injury to the central nervous system (CNS), whether metabolic, structural, auto-immune, ischemic, infectious or mechanical, results in increased production of pro-inflammatory cytokines such as TNF, IL-1β and IL-6, and of neurotoxic molecules such as nitric oxide (NO), by activated microglia and astrocytes. Such pro-inflammatory environment culminates in cell death and cerebral tissue damage [7,8].

Anti-inflammatory therapies including IL-1 receptor antagonist, IL-1β inhibitors and NSAIDs have shown some benefit in reducing post-stroke neurodegenerative lesions, and in decreasing incidence of AD or PD [9-11]. However, widespread use of these therapies must be cautioned by their inhibitory effect on NFκB, a transcription factor that regulates expression of many pro-inflammatory mediators, all the while promoting the upregulation of protective and regenerative molecules in the CNS [12]. Therefore, a better understanding of the molecular signature of neuroinflammatory diseases is required in order to identify safe, effective and possibly disease-specific therapeutic targets.

A20 (TNF alpha-induced protein 3, *TNFAIP3*), a pleiotropic NFκB-dependent gene [13] expressed in a variety of tissues and cell types, including the human brain [14], encodes a ubiquitin-editing enzyme that is essential for termination of NFκB activation in response to multiple stimuli such as IL-1β, TNF, IL-6, CD40 and lipopolysaccharide (LPS) [15-17]. The potent anti-inflammatory role of A20 is exemplified by the phenotype of A20 knockout (KO) mice. These mice rapidly become cachectic and die within three to five weeks of age, as a result of uncontrolled inflammation in several organs [18]. In contrast to its ubiquitous anti-inflammatory function, A20’s effect on apoptosis is cell type specific. A20 exhibits potent anti-apoptotic properties in endothelial cells (EC), hepatocytes and pancreatic β-cells, through several mechanisms including blockade of the caspase cascade at the level of caspase 8, and preservation of mitochondrion integrity [19]. On the other hand, A20 promotes vascular smooth muscle cells apoptosis through a NO-dependent mechanism [20]. In addition, overexpression of A20 protects livers and kidneys from ischemia reperfusion injury, in part by upregulating peroxisome proliferator associated receptor alpha, channeling lipid metabolism away from lipid peroxidation towards mitochondrial β-oxidation, which results in increased ATP generation [21,22].

Little is known about the role of A20 in the CNS. Evidence from the literature suggest that increased expression of A20 is neuroprotective in animal models of epilepsy [23] and of focal cerebral ischemia, in part by limiting ischemic damage and containing TNF-induced neuronal apoptosis [24]. However, these gain-of-function studies did not address the physiological role of A20 nor its involvement in maintaining homeostasis and, in particular, containing inflammation in the CNS. In more recent studies, mice with neuroectodermal (astrocytes, neurons and oligodendrocytes) specific A20 KO failed to show increased CNS inflammation at baseline [25] and did not demonstrate larger ischemic infarcts following middle cerebral artery occlusion [26], when compared to their wild type (WT) littermates. However, CNS specific A20 KO did develop an aggravated form of auto-immune encephalomyelitis, which was attributed to astrocytic loss of A20, further fueling controversy about A20’s function in the CNS [25]. In order to better delineate the physiological role of A20 in modulating CNS inflammation, we phenotyped the cerebral inflammatory pattern of full A20 KO and heterozygous (HT) mice. We reasoned that this approach carries greater clinical relevance than cell type-specific KO of this gene, as it would enable us to gauge the impact of A20 on CNS generators of inflammation (microglial cells, astrocytes), as well as CNS targets of inflammation (neurons, endothelial cells). This study is timely given the many A20 single SNPs causing decreased expression or function of this gene that have been associated with numerous auto-immune and inflammatory diseases [25,27-30].

### Experimental procedures

Reagents: Human recombinant TNF was purchased from R&D Systems (Minneapolis, MN, USA). LPS (Lipopolysaccharides from *Escherichia coli* 055:B5), and FBS were obtained from Sigma-Aldrich Co. (St. Louis, MO, USA).

### Mice

Four to five-week-old A20 KO, HT and WT littermate control mice [18] were used for forebrain isolation. Following anesthesia, mice were sacrificed by decapitation, and their brains recovered and fixed for analysis by immunohistochemistry (IHC) and immunofluorescence (IF). Alternatively, cerebral cortex (CX) and hippocampus (HC) were isolated for mRNA and protein isolation. For primary astrocyte isolation and culture, brains from one to three-day-old pups were used. Animals received humane care according to the criteria outlined in the Guide for the Care and Use of Laboratory Animals. Beth Israel Deaconess Medical Center Institutional Animal Care and Use Committee approved all research protocols.

### Cell culture

Primary astrocytes were prepared from forebrain of neonatal mice (one to three-day-old) according to a modified method of McCarthy and De Vellis [31]. Purity of astrocyte preparation was > 95%. In brief, cerebral hemispheres were freed from the meninges and the forebrain was dissociated mechanically using fire-polished Pasteur pipets. Mixed brain cells were plated in DMEM containing 10% FBS, and antibiotics (Mediatech, Inc., Manassas, VA, USA). Cells were cultured for seven to ten days until confluent in a humidified atmosphere enriched with 5% CO_2_. Contaminating oligodendrocytes and microglial cells were eliminated from the astrocytic monolayer by placing culture flasks on a rotary shaker at 800 rpm overnight. Astrocyte monolayers were then trypsinized and cells plated in 24-well plates and cultured to confluency for seven to ten days before being used in experiments. The mouse microglia cell line N13 (kind gift of Dr. Di Virgilio, University of Ferrara, Italy) and mouse primary astrocytes purchased from ScienCell Research Laboratories (Carlsbad, CA, USA) were used in RNA silencing experiments.

### Western blot

Tissue lysates (40 to 60 μg protein) were separated under reducing conditions by SDS-PAGE (Bio-Rad Laboratories, Hercules, CA, USA) [32], and transferred to Polyvinylidene fluoride (PVDF) membranes (PerkinElmer Life Science, Whaltham, MA, USA) by semi-dry electroblotting. Membranes were probed with mouse anti-gp91^phox^ (BD Pharmigen, San Diego, CA, USA), mouse anti-glyceraldehyde 3-phosphate dehydrogenase (GAPDH) (EMD chemicals), mouse anti-βactin and rabbit anti-IκBα (Santa Cruz Biotechnology, Inc., Santa Cruz, CA, USA). Appropriate secondary horseradish peroxidase (HRP)-conjugated antibodies were used (Thermo Scientific, Rockford, IL, USA). Protein bands were detected with enhanced chemiluminescence kit (ECL) (PerkinElmer Life Science, Waltham, MA, USA) followed by exposure to the autoradiography film. Immunoblots were scanned and the intensity of the bands was quantified by densitometry using ImageJ 1.41 (US National Institutes of Health, Bethesda, MD, USA).

### Silencing RNA (siRNA)

N13 microglia cells and mouse primary astrocytes (ScienCell Research Laboratories, Carlsbad, CA, USA) were transfected with predesigned A20 silencing RNA probes (A20 siRNA) or All Start Negative Control siRNA (C siRNA), using Hiperfect transfection reagent purchased from Qiagen (Valencia, CA, USA). Transfections were carried out according to the manufacturer’s transfection protocol. Experiments were performed 24 hours after transfection. Efficiency of gene knockdown was evaluated by qPCR in non-treated and LPS (1 μg/mL for 1 hour) treated cells.

### TNF and IL-6 secretion

Cell culture medium was changed to serum-free medium, then cells were stimulated with either TNF or LPS in order to mimic inflammation. Supernatants were then recovered, and analyzed for IL-6 and TNF content by ELISA, using mouse IL-6 and TNF ELISA Ready-SET-Go! (eBioscience, San Diego, CA, USA), according to manufacturer’s instruction. Results were normalized by protein content. Cell cultures incubated in medium alone were used as non-stimulated controls.

### Quantitative reverse transcriptase-polymerase chain reaction (qPCR)

mRNA from CX and HC and from primary astrocytes and the mouse microglia cell line N13 was isolated using RNAse spin columns (Qiagen, Valencia, CA, USA), and cDNA was synthesized using iScript cDNA synthesis kit (Bio-Rad, Hercules, CA, USA). Real-time PCR (qPCR) was performed using iTaq Fast SYBR Green Supermix with ROX (Bio-Rad, Hercules, CA, USA) and gene specific primers (Table 1) or TaqMan Mm00627280_m1 (tnfaip3), Mm00607939_s1 (βactin), and ABI 7500 Fast Real-time PCR System (Applied Biosystems, Inc., Foster City, CA, USA). Comparative threshold cycle (Ct) method was used to perform relative quantification of qPCR results. mRNA expression of target genes A1, TNF, IL-6, inducible NOS (iNOS), endothelial NO synthase (eNOS), neuronal NO synthase (nNOS), E-selectin, monocyte chemoattractant protein 1 (MCP-1), glial fibrillary acidic protein (GFAP), nuclear factor erythroid 2 related factor 2 (Nrf2), IκBα, gp91^phox^ and heme oxygenase-1 (HO-1) was normalized to that of the housekeeping gene βactin. Data are expressed as fold change of levels noted in WT mice.

**Table 1:** List of primers used in real-time PCR

**Gene**	**Definition**	**Accession**	**Forward**	**Reverse**
IL-6	*Mus musculus* interleukin 6	NM_031168.1	GACAACCACGGCCTTCCCTACTTC	TCATTTCCACGATTTCCCAGAGA
TNF	*Mus musculus* tumor necrosis factor	NM_013693.2	GACAAGGCTGCCCCGACTACG	CTTGGGGCAGGGGCTCTTGAC
iNOS	*Mus musculus* nitric oxide synthase 2, inducible (Nos2)	NM_010927.3	AACAGAGCCCTCAGCAGCATCCAT	CCAGGTGTTCCCCAGGCAGGTAG
eNOS	*Mus musculus* nitric oxide synthase 3, endothelial cell (Nos3)	NM_008713.4	TCACTTCGTTCGGTTGACCA	CCTTCAAGATTTAGGCCGACCC
nNOS	*Mus musculus* nitric oxide synthase 1, neuronal (Nos1)	NM_008712.2	GCCGCCAAAACCTGCAAAGTCCTA	CGCGTCCTCCAGCCGTTCAAT
GFAP	*Mus musculus* glial fibrillary acidic protein (Gfap), transcript variant 1 and variant 2	NM_001131020.1 NM_010277.3	TACCATGCCACGCTTCTCCTTGTC	ACGCTCGCTCGCCCGTGTCTCCT
E-selectin	*Mus musculus* selectin, endothelial cell (Sele)	NM_011345.2	CTTGACGTCCCGGGAAAGATGAAC	GGGACGGGTGGGGCTGACTGG
MCP-1	*Mus musculus* chemokine (C-C motif) ligand 2 (Ccl2)	NM_011333.3	GTTAACGCCCCACTCACCT	AAAAACTACAGCTTCTTTGGGACACCT
A1	*Mus musculus* B-cell leukemia/lymphoma 2 related protein A1a (Bcl2a1a)	NM_009742.3	TGGGGGTGTTCTCCTCAAAAAA	AAGCCATCTTCCCAACCTCCATTC
IL-1β	*Mus musculus* interleukin 1 beta	NM_008361.3	AAATCTCGCAGCAGCACATCAA	CCACGGGAAAGACACAGGTAGC
Nrf2	*Mus musculus* nuclear factor, erythroid derived 2, like 2 (Nfe2l2)	NM_010902.3	CCGGCCCAGCACATCCAGACAGAC	GGGATATCCAGGGCAAGCGACTCA
Gp91phox	*Mus musculus* cytochrome b245, beta polypeptide (Cybb)	NM_007807.5	CAAGTGCCCCAAGGTATCCAAGTT	TGAATAGCCCCTCCGTCCAGTCTC
HO-1	*Mus musculus* heme oxygenase (decycling) 1 (Hmox1)	NM_010442.2	GCCCACGCATATACCCGCTACCT	CCATGGCCTTCTGTGCAATCTTCT
IκBα	Nuclear factor of kappa light polypeptide gene enhancer in B-cells inhibitor, alpha	NM_010907	CTACACCTTGCCTGTGAGCA	TCCTGAGCATTGACATCAGC

### Immunohistochemistry (IHC) and immunofluorescence (IF)

Brains were processed for IHC and IF as described [33]. In brief, 2,000-μm coronal slices were zinc-fixed (BD Pharmigen, San Diego, CA, USA) for 48 hours at room temperature, dehydrated in a tissue processor and embedded in paraffin for sectioning, before being sectioned into 6-μm thickness. For IHC, sections were de-paraffinized, rehydrated, fixed with cold acetone:formalin 95:5 (vol/vol) for 3 minutes, then incubated with horse serum (7% in PBS) prior to overnight incubation at 4°C with hamster anti-ICAM-1 and rat anti-VCAM-1 (BD Pharmigen, San Diego, CA, USA), rabbit anti-iNOS and anti-IL-6 (Abcam Inc., Cambridge, MA, USA), and rabbit anti-GFAP (Dako, Carpinteria, CA, USA). Alternatively, sections were fixed with cold 2% paraformaldehyde for 10 minutes prior to overnight incubation with mouse anti-nitrotyrosine (Santa Cruz Biotechnology, Inc., Santa Cruz, CA, USA), rat anti-F4/80 (AbDSerotec, Raleigh, NC, USA), rabbit anti-TNF alpha (Novus Biologicals, Littleton, CO) and hamster anti-MCP-1 (BD Pharmigen, Franklin Lakes, NJ, USA). Sections were then treated with H_2_O_2_ 1:100 in PBS for 10 minutes, incubated with the appropriate secondary IgG antibodies followed by ABC (avidin-biotin complex) reagent (Vector Laboratories, Burlingame, CA, USA), then detected by ImmPACT 3,3’-diaminobenzidine tetrahydrochloride (DAB) peroxidase substrate (Vector Laboratories, Burlingame, CA, USA). Negative controls using only secondary antibodies confirm the absence of non-specific immunostaining (Additional file 1: Figure S1). For IF, sections were fixed with 2% cold paraformaldehyde for 10 minutes prior to overnight incubation with rabbit anti-Nrf2 (Abcam plc., Cambridge, MA, USA), rabbit anti-IL-6, rat anti-F4/80 and goat anti-GFAP (Santa Cruz, Biotechnology, inc., Dallas, TX, USA), followed by appropriate Alexa Fluor 488 (green) and 594 (red) conjugated secondary antibodies (Invitrogen, Carlsbad, CA, USA).

### Statistical analysis

Results are presented as mean ± standard error of mean (SEM). Statistical analysis was performed on Prism 5 for Mac (GraphPad Software, Inc., La Jolla, CA, USA). Data were analyzed by one or two way analysis of variance (ANOVA) followed by *post-hoc* Tukey or Bonferroni, respectively, when F was significant. Alternatively, results were analyzed by a non-parametric Kruskal-Wallis analysis followed by Dunn’s multiple comparison tests when variances differed significantly. Differences between groups were rated significant at a probability error (*P*) < 0.05.

## Results

### Baseline A20 mRNA expression and NFκB activation in mouse cerebral cortex and hippocampus

**Figure 1:**
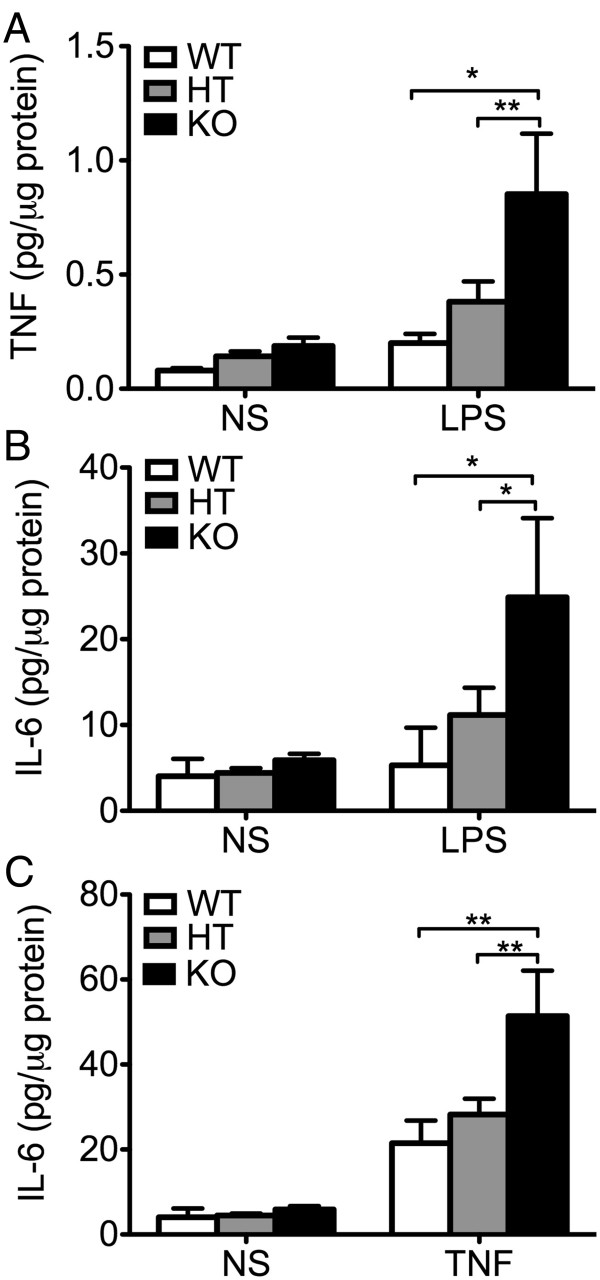
**Baseline A20 mRNA expression in mouse cerebral cortex and hippocampus. (A)** A20 and **(C)** IκBα mRNA levels in cerebral cortex (CX) and hippocampus (HC) of wild type (WT), A20 heterozygous (HT) and A20 knockout (KO) mice, measured by qPCR. Graph shows relative mRNA levels after normalization with mRNA levels of housekeeping gene βactin. Results are expressed as mean ± SEM of three to four animals per genotype. **P < 0.01. ND: not detectable. **(B)** Western blot (WB) analysis of IκBα expression in CX and HC of A20 WT, HT and KO mice. Immunoblotting for the housekeeping protein βactin was used to control for loading. Results are representative of three animals per genotype.

We first probed for expression and distribution of A20 in normal and A20 deficient mouse brain. By qPCR, we demonstrated that A20 mRNA was detected in comparable amounts in CX and HC of WT mice (Figure 1A). As expected, A20 mRNA was undetectable in the brain of KO mice and showed 50% reduction in the brain of HT mice (Figure 1A). A20 wipeout correlated with higher NFκB activation in cerebral cortex and hippocampus of KO versus WT mice, as evidenced by remarkably lower IκBα protein levels, indicative of amplified degradation (Figure 1B) while IκBα protein levels were intermediate in CX and HC of HT mice. Corroborating heightened NFκB activation in the brains of A20 KO mice, we noted significantly higher mRNA levels of IκBα, itself a prime NFκB-dependent gene, when compared to WT brains, with again HT brains showing intermediate results (Figure 1C).

### Total and partial loss of A20 causes reactive microgliosis and astrogliosis

Having confirmed absence and decreased expression of A20 in brains of KO and HT mice, respectively, we evaluated the impact of total or partial loss of A20 on microglia and astrocytes activation. Microglia, the resident macrophages of the CNS, and astrocytes, the most abundant glial cell population, respond to injury and inflammation by assuming an activated phenotype defined by characteristic changes in morphology and gene expression, and by increased propensity for migration and proliferation [34,35].

**Figure 2:**
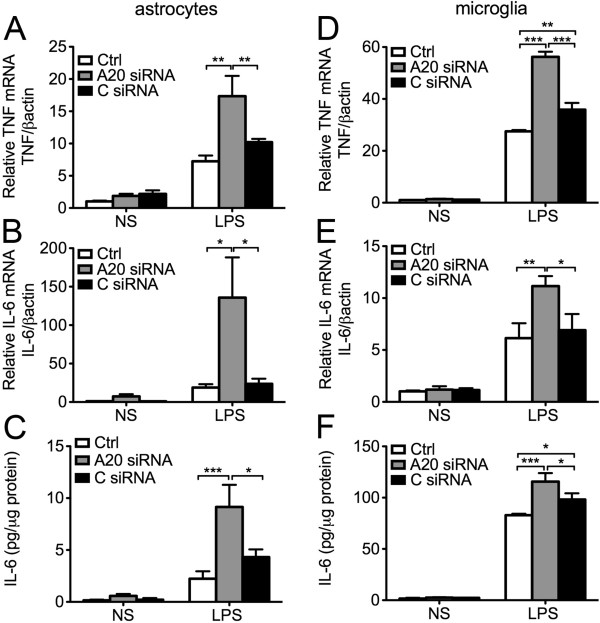
**Loss of A20 leads to spontaneous microglia and astrocyte activation.** Representative **(A)** F4/80 and **(C)** GFAP immunohistochemistry (brown) in the cerebral cortex (CX) and hippocampus (HC) of A20 wild type (WT), heterozygous (HT) and knockout (KO) mice. Yellow arrows indicate hypertrophied activated microglia, noted by their stout, dense appearance with shorter and thicker branched projections. Blue arrows indicate reactive astrocytes displaying thick cell bodies and processes, evident in the outer layers of the CX and throughout the HC. Photomicrographs are representative of three animals per genotype. Bar = 20 μm, magnification = 400x. **(B)** A1 and **(D)** GFAP mRNA levels measured by qPCR. Graph shows relative mRNA levels after normalization with mRNA levels of housekeeping gene βactin. Results are expressed as mean ± SEM of five to seven animals per genotype. *P < 0.05, **P < 0.01 and ***P < 0.001.

Immunohistochemistry analysis of mouse brain sections using a macrophage/microglia cell surface marker F4/80 [36] revealed increased number of activated microglia throughout the A20 KO brain, as evidenced by their typical hypertrophied phenotype, that is enlarged cells with shorter and thicker branched processes [37] (Figure 2A). This picture totally contrasted with WT brains that showed resting/quiescent microglia harboring a ramified morphology with slender sensing arms. We confirmed the activation status of microglia by probing for mRNA levels of the microglial activation marker A1 [38,39]. A1 mRNA levels were significantly higher in CX and HC (approximately seven-fold) of A20 KO as compared to WT mice (Figure 2B). Astrocyte activation in the brain of A20 KO mice was also evident, as demonstrated by enhanced GFAP immunoreactivity [40]. Astrocytes displaying thick cell bodies and processes characteristic of astrocyte reactivity were especially marked in the outer layers of the CX and throughout the HC (Figure 2C). Astrocyte activation was confirmed at the mRNA levels by qPCR. GFAP mRNA levels were significantly (approximately 1.8-fold) higher in the CX and HC of A20 KO mice as compared to WT (Figure 2D). Brains from HT mice showed an intermediate phenotype with a consistent trend for greater microglia and astrocyte activation when compared to WT mice, and for significantly lower microglia and astrocyte activation when compared to KO mice, as showed by IHC and qPCR (Figure 2).

### Total and partial loss of A20 increases brain cytokine and chemokine levels

**Figure 3:**
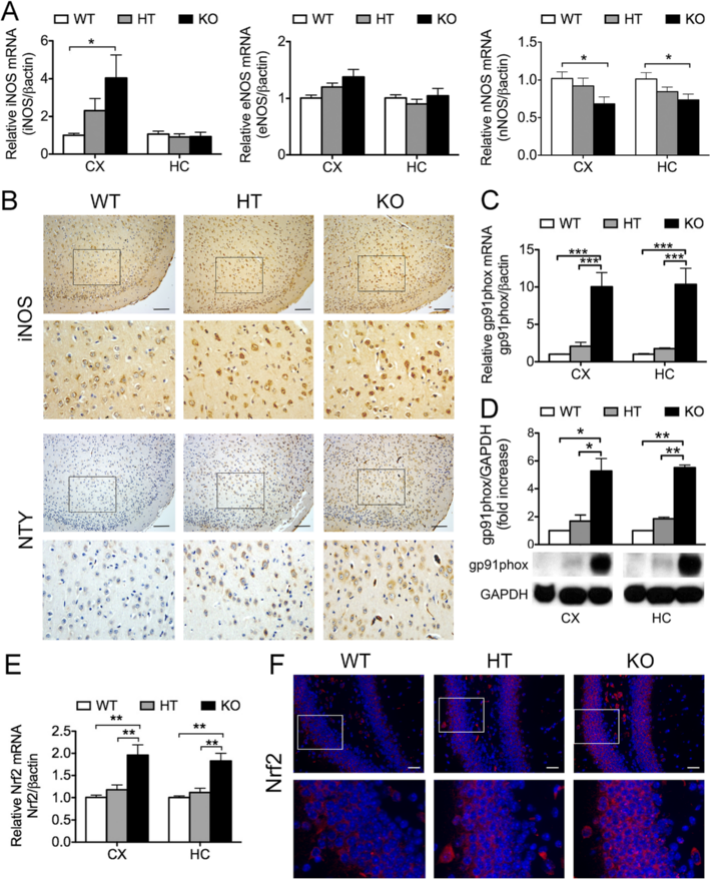
**Levels of pro-inflammatory mediators are increased in cerebral cortex and hippocampus of A20 deficient mice. (A)** TNF, IL-6, IL-1β and MCP-1 mRNA levels in cerebral cortex (CX) and hippocampus (HC) of wild type (WT), A20 heterozygous (HT) and A20 knockout (KO), measured by qPCR. Graph shows relative mRNA levels after normalization with mRNA levels of housekeeping gene βactin. Results are expressed as mean ± SEM of four to seven animals per genotype. *P < 0.05, **P < 0.01 and ***P < 0.001. **(B)** Representative images of TNF, IL-6 and MCP-1 immunohistochemistry (brown) in HC (TNF and IL-6) and CX (MCP-1) of A20 WT, HT and KO mice. Photomicrographs are representative of three to four animals per genotype. Top images: Bar = 50 μm, magnification = 200x. Bottom images are close-up images of the area delineated by the black box in top images.

**Figure 4:**
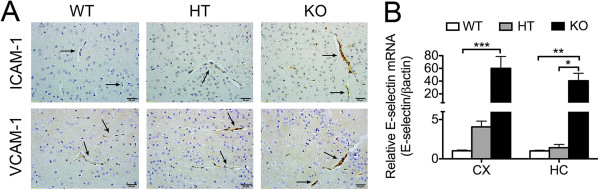
**Astrocytes and microglia contribute to higher IL-6 levels in A20 deficient brains.** Representative images of double IL-6 (red) and GFAP (green), or IL-6 (red) and F4/80 (green) positive cells in the hippocampus (HC) of A20 wild type (WT), heterozygous (HT) and knockout (KO) mice, as determined by immunofluorescence staining. White arrows show IL-6 co-localization with glial fibrillary acidic protein (GFAP) (astrocytes) or F4/80 (microglia), as evidenced by the yellow overlay. Nuclei were stained with 4′,6-diamidino-2-phenylindole (DAPI, blue). Photomicrographs are representative of four animals per genotype. Top images: Bar = 20 μm, magnification = 400x. Bottom images are close-up images of the area delineated by the white box in top images.

Activated microglia and reactive astrocytes are key defense mechanisms of the CNS to injury, in part through their ability to modulate immune and inflammatory responses by secreting pro-inflammatory cytokines and chemokines such as TNF, IL-6, IL-1β, and MCP-1 [41,42]. As in all inflammatory responses, this defense system needs to be tightly modulated in order to avoid unfettered inflammation that would counterproductively cause neurotoxicity [43]. Accordingly, we probed by qPCR for mRNA levels of TNF, IL-6, IL-1β and MCP-1 in CX and HC of A20 WT, HT and KO mice. Our results show significantly increased mRNA levels of all these pro-inflammatory mediators in the brain of KO, as compared to WT mice, further confirming glial activation (Figure 3A). HT mouse brains also showed a tendency (albeit not significant) for higher mRNA levels of all these molecules when compared to WT brains. This tendency was more prominent in the CX than in the HC. We confirmed by IHC that higher TNF, IL-6, and MCP-1 mRNA levels in the brain of A20 deficient mice correlated with higher protein levels (Figure 3B). Double immunofluorescence staining using antibodies against microglia surface marker F4/80 or astrocyte marker GFAP in combination with anti-IL-6 demonstrate that both cell types produce IL-6 and contribute to its increased levels in the brains of A20 KO mice (Figure 4). Altogether, these results indicate a heightened basal level of inflammation in the brain of A20 deficient mice, especially when A20 expression is totally knocked-out.

### Total and partial loss of A20 increases cytokine production by primary astrocytes and microglia cell line in response to pro-inflammatory stimuli

**Figure 5:**
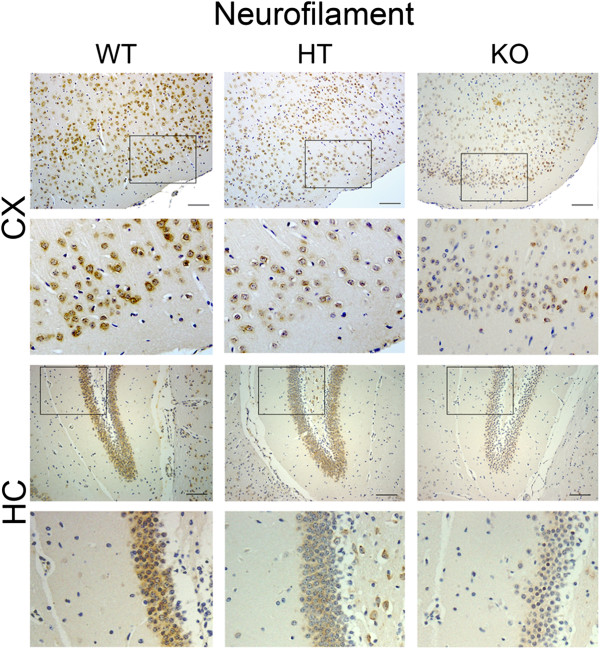
**Cytokine production in response to inflammatory stimuli is enhanced in A20 deficient primary astrocytes. (A)** TNF and **(B)** IL-6 levels, measured by ELISA, in cell culture supernatant from A20 wild type (WT), heterozygous (HT) and knockout (KO) mouse primary astrocytes following 24 hour stimulation with LPS (10 μg/mL). **(C)** IL-6 levels measured by ELISA, in cell culture supernatant from WT, HT and KO mouse primary astrocytes following 24 hours stimulation with TNF (100 UI/mL). NS: non-stimulated cells. Data represent mean ± SEM of primary astrocytes isolated from littermate pups (WT n = 2; HT n = 8 to 10, KO n = 3 to 4). *P < 0.05, **P < 0.01.

**Figure 6:**
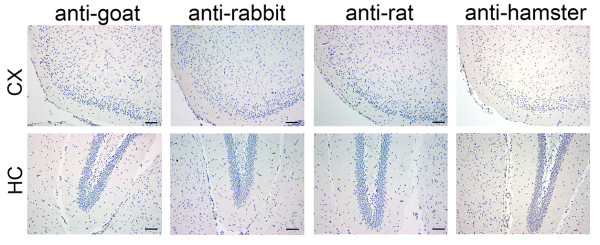
**A20 knockdown enhances lipopolysaccharide (LPS)-mediated cytokine production in mouse primary astrocytes and microglia cell line. (A)** TNF and **(B)** IL-6 mRNA levels in mouse primary astrocytes 6 hours after LPS (1 μg/mL) stimulation, as measured by qPCR. **(D)** TNF and **(E)** IL-6 mRNA levels in the microglia cell line N13, 1 hour and 6 hour respectively, after LPS (1 μg/mL) stimulation, as measured by qPCR. Graph shows relative mRNA levels after normalization with mRNA levels of housekeeping gene βactin. IL-6 protein levels, measured by ELISA, in cell culture supernatant of **(C)** mouse primary astrocytes and **(F)** microglia cell line N13 6 hours after LPS (1 μg/mL) stimulation. Results are expressed as mean ± SEM of three to five independent experiments. NS: non-stimulated cells. Ctrl: non-transfected control cells. A20 siRNA: cells transfected with A20 silencing RNA. C siRNA: cells transfected with All Star control silencing RNA. *P < 0.05, **P < 0.01 and ***P < 0.001.

IL-6, mostly produced by astrocytes, achieves higher concentration than other soluble pro-inflammatory mediators in the brain, and hence has been designated as a key contributor to neuroinflammation [44,45]. To check whether enhanced IL-6 levels in the brain of A20 KO mice resulted from heightened activators, that is higher TNF levels, or also related to heightened production by A20 deficient astrocytes in response to a similar level of activator, we isolated and cultured primary astrocytes from A20 KO, HT and WT mice, exposed them for 24 hours to similar concentrations of exogenous LPS (10 μg/mL) and measured, by ELISA, TNF and IL-6 levels in cell culture supernatant. We noted a trend towards higher (albeit not significant) TNF and IL-6 levels in 24 hours culture supernatants of A20 KO as compared to WT astrocytes (2.25- and 1.5-fold, respectively), in the absence of any inflammatory stimuli. Following LPS treatment, TNF and IL-6 levels increased in response to LPS in all groups, albeit these levels were significantly higher in KO, as compared to WT astrocytes (Figure 5A and B). HT astrocytes showed an intermediate response, that is LPS treatment increased IL-6 production by two to three-fold, as compared to a ten-fold upregulation in KO astrocytes (Figure 5B). Since higher LPS-induced TNF levels is the master inducer of IL-6 in astrocytes, and hence could account for higher IL-6 levels in LPS treated A20 deficient astrocytes, we independently evaluated TNF-induced production (100 U/ml) of IL-6 in these cells. Here again, TNF-induced upregulation of IL-6 production was significantly higher in A20 KO as compared to WT and HT astrocytes (Figure 5C). We confirmed these findings in mouse primary astrocytes that had undergone siRNA mediated A20 knockdown. Transfection of astrocytes with A20 siRNA reduced by 50% LPS-induced A20 upregulation, as evidenced by mRNA levels measured 1 hour after LPS (1 μg/mL) stimulation (Additional file 2: Figure S2). Inadequate A20 upregulation following LPS (mimicking A20 knockdown in A20 HT mice) correlated with significantly higher TNF and IL-6 mRNA levels six hours after LPS, as compared to levels measured in non-transfected and All Star siRNA (C siRNA) control cells (Figure 6A and B). This was paralleled by significantly higher IL-6 protein levels in the cell culture supernatant of A20siRNA versus control cells (Figure 6C). As in astrocytes, siRNA mediated A20 knockdown in microglia cells (N13) decreased by 50% LPS induced upregulation of A20 (Additional file 2: Figure S2). Here again, this correlated with significantly higher LPS-induced upregulation of TNF and IL-6 mRNA (Figure 6D and E), and of IL-6 protein (Figure 6F) levels in these cells as compared to non-transfected or C siRNA transfected cells.

Altogether these results establish that both heightened activators (TNF) in brains of A20 deficient mice, and hyper-responsiveness of A20 deficient glia to pro-inflammatory stimuli contribute to the amplification of the pro-inflammatory spiral, culminating in excessive amount of IL-6.

### Total and partial loss of A20 increases brain oxidative and nitrosative stress

**Figure 7:**
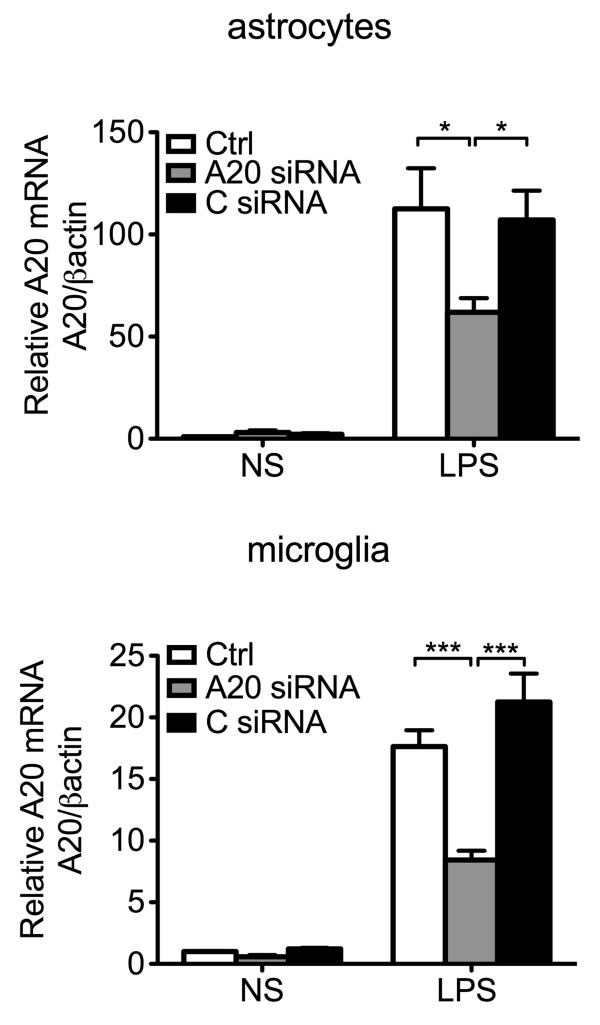
**Loss of A20 increases oxidative/nitrosative stress in the brain. (A)** iNOS, eNOS and nNOS, **(C)** NADPH oxidase gp91^phox^ subunit and **(E)** Nrf2 mRNA levels in cerebral cortex (CX) and hippocampus (HC) of wild type (WT), A20 heterozygous (HT) and A20 knockout (KO) mice, measured by qPCR. Graphs show the statistical analysis of relative mRNA levels after normalization with βactin. Results are expressed as mean ± SEM of five to seven animals per genotype. **(B)** iNOS and nitrotyrosine (NTY) immunostaining (brown) in CX of A20 WT, HT and KO mice. Top images: Bar = 50 μm, magnification = 200x. Bottom images are close-up images of the area delineated by the black box in top images. **(D)** NADPH oxidase gp91^phox^ subunit expression in CX and HC protein lysates of A20 WT, HT and KO brains evaluated by Western blot (WB). Housekeeping protein GAPDH was used as loading control for semi-quantitative densitometry as shown in the graph. Graph shows semi-quantitative densitometry data using GAPDH as loading control. Results are expressed as mean ± SEM for four animals per genotype. **(F)** Representative images of Nrf-2 (red) and 4′,6-diamidino-2-phenylindole (DAPI, nuclear staining, blue) immunofluorescence staining in HC of WT, HT and KO mice. Photomicrographs are representative of three animals per genotype. Top images: Bar = 50 μm, magnification = 200x. Bottom images are close-up images of the area delineated by the white box in top images.*P < 0.05 and **P < 0.01.

NO, when produced in physiologic levels by the low-throughput and constitutively expressed nNOS and eNOS NO synthases (NOS), mostly serves a homeostatic function through regulation of synaptic signaling and plasticity [46,47], as well as vasoprotection, through combined anti-apoptotic, anti-inflammatory, and reactive oxygen radicals’ scavenging properties [48]. However, overproduction of NO in pathophysiological conditions is implicated in oxidative-dependent neuronal death and dysfunction. High throughput NFκB-dependent iNOS, mainly produced by activated glia, is the primary NOS involved in inflammatory neurodegenerative disorders [49]. Accordingly, we evaluated iNOS mRNA and protein levels in CX and HC of A20-competent and A20 deficient mice. Our results show that iNOS mRNA levels significantly increase in the CX of KO mice as compared to WT, with HT demonstrating an intermediate level (Figure 7A). We confirmed this by IHC that depicted increased iNOS immunostaining in the CX of A20 deficient mice (Figure 5B). Levels of iNOS mRNA in HC were similar in all groups. Besides high NO production by iNOS, drastic increase of nNOS expression in certain pathophysiologic conditions could promote excitotoxicity causing neuronal death [50]. Interestingly, nNOS mRNA levels were significantly decreased in CX and HC of A20 KO, as compared to WT mice, with HT showing an intermediate phenotype (Figure 7A), while brain eNOS mRNA levels were comparable in all three genotypes (Figure 7A). Increased expression of iNOS in inflammatory conditions is often associated with increased levels of NADPH oxidase, the major enzymatic complex involved in the production of superoxide anion (O_2_-) [51]. Increased NO levels, in the setting of oxidative stress, favors formation of highly reactive peroxynitrite (NO/O_2_) species, enhancing formation of the protein adduct, nitrotyrosine [52]. Accordingly, we evaluated, by qPCR and Western blot, the expression level of the transmembrane catalytic subunit of NADPH oxidase, gp91^phox^. Our results show that gp91^phox^ mRNA and protein levels were significantly higher in CX and HC of A20 KO as compared to WT and HT mice, with HT mice showing slightly higher levels then their WT littermates (Figure 7C and D). Combined increase of gp91^phox^ and iNOS (hence likely NO) production in brains of A20 KO mice correlated with increased immunostaining for nitrotyrosine, indicating heightened levels of protein nitration, implying nitrosative stress (Figure 7B).

Oxidative stress in the brain is also regulated by the down-modulating effect of the transcription factor Nrf2. Nrf2 is activated in response to oxidative stress, and initiates the transcription of several antioxidant and cytoprotective genes [53,54]. Nrf2 also antagonizes inflammation in the brain by negatively impacting NFκB activation [55]. Accordingly, we checked whether A20 knockdown also increases inflammation and oxidative stress in the brain through impacting Nrf2 expression or function. Interestingly, our studies show increased Nrf2 mRNA and protein levels in A20 KO brains as compared to control (Figure 7E and F), possibly an attempt to contain oxidative stress. However, increased Nrf2 levels did not translate into any significant induction of Nrf2-dependent cytoprotective and antioxidant genes such as HO-1 in A20 KO brains, suggesting that A20 knockdown likely interfered with Nrf2 activation, precluding an adequate regulatory antioxidant response in these mice (Additional file 3: Figure S3). Altogether, our results indicate that heightened inflammation in the brain of A20 deficient mice associates with enhanced oxidative and nitrosative tissue damage.

### Endothelial cell activation is increased in brain of A20 deficient mice

**Figure 8:**
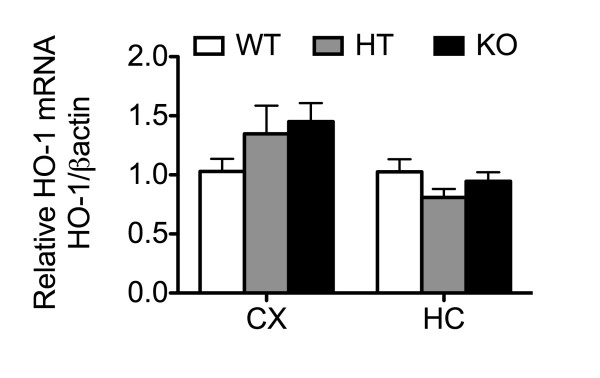
**Expression of adhesion molecules is increased in A20 deficient brain vasculature. (A)** ICAM-1 and VCAM-1 immunohistochemistry (brown) in cerebral cortex (CX) of A20 wild type (WT), heterozygous (HT) and knockout (KO) mice. Black arrows indicate blood vessels. Photomicrographs are representative of three animals per genotype. Bar = 20 μm, magnification = 400x. **(B)** E-selectin mRNA levels in CX and hippocampus (HC) of A20 WT, HT and KO mice, measured by qPCR. Graph shows the statistical analysis of relative mRNA levels after normalization with βactin. Results are expressed as mean ± SEM for four to six animals per genotype. *P < 0.05, **P < 0.01 and ***P < 0.001.

Activation of brain EC and subsequent upregulation of adhesion and other pro-inflammatory molecules is an obligate corollary of heightened cerebral inflammation and oxidative stress, as noted in A20 KO mice [56]. Indeed, we confirmed that mRNA levels of the EC specific and prototypic activation marker, the adhesion molecule E-selectin, were significantly increased in CX and HC of A20 KO mice, as compared to WT, with HT fairing in between (Figure 8A). Similarly, vascular immunostaining for the adhesion molecules ICAM-1 and VCAM-1 was much stronger in brain sections of A20 KO mice, as compared to the faint staining observed in WT mice, with HT mice showing an intermediate staining (Figure 8B). These results demonstrate that A20 deficiency also caused spontaneous basal endothelial cell activation in the brain. Inflammation may results in disruption of the blood brain barrier (BBB), which allows for increased cytokine access to the brain. To evaluate whether permeability of the BBB was affected in A20 deficient mice, we intravenously injected a 2% Evan’s blue dye solution to A20 WT, HT and KO and measured the amount of dye that extravasated into the brain parenchyma. We also evaluated the integrity of the BBB by measuring serum levels of S100 calcium-binding protein β (S100β), an astrocyte molecule usually released into the peripheral circulation upon disruption of these cells’ membrane integrity, and a good indicator of enhanced BBB permeability [57]. Our results demonstrated that the integrity of the BBB was not altered in A20 deficient brains, despite their higher basal inflammation and EC activation levels (Additional file 4: Figure S4, Additional file 5: Supplementary Experimental Procedures).

### Total and partial loss of A20 promotes axonal injury

**Figure 9:**
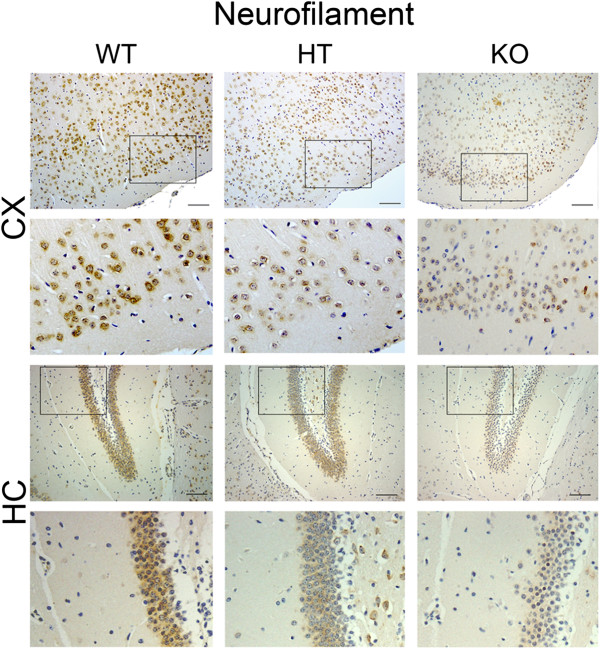
**Loss of A20 decreases the expression of neurofilaments in mice cerebral cortex and hippocampus.** Immunostaining for neurofilaments (NF) (brown) in cerebral cortex (CX) and hippocampus (HC) of wild type (WT), A20 heterozygous (HT) and A20 knockout (KO) mice. Photomicrographs are representative of three animals per genotype. Top images: Bar = 50 μm, magnification = 200x. Bottom images are close-up images of the area delineated by the black box in top images.

Neurofilaments (NF) are intermediate filaments of the cytoplasmic scaffold that composes the axon cytoskeleton [58]. Expression of NF proteins decreases in physiological and pathological conditions such as aging [59], AD [60], amyotrophic lateral sclerosis and MS. Accordingly, NF protein expression levels qualify as surrogate for neuronal response to injury. Despite the fact that reduction in NF proteins is generally well tolerated [59], it is associated with decreased axonal transport velocity [61]. Brains of A20 KO mice showed significantly less immunostaining for NF (Figure 9), as compared to WT mouse brain. This is suggestive of axonal damage likely induced by chronic neuronal exposure to a pro-inflammatory environment. A20 HT mice showed an intermediate phenotype.

## Discussion

A20 is a ubiquitously expressed NFκB negative feedback regulator that is highly and rapidly induced in response to NFκB activation in most cell types and organs, including the human brain [25,26]. We demonstrated basal A20 mRNA expression in the mouse CX and HC, the two brain structures that were the focus of this study, though the comparative distribution of A20 in the different brain regions varies between mice and humans [14]. We verified that basal A20 mRNA levels in CX and HC decreased by half and were totally absent in brains of A20 HT and KO, respectively. Lower A20 levels correlated with higher NFκB activation in the brains of A20 KO mice.

The inflammatory phenotype of A20 KO mice, which are cachectic and die prematurely due to uncontrolled spontaneous inflammation in the liver, kidney, joints, intestines and bone marrow, has been previously characterized [18]. However, it lacked an account of the impact of A20’s deficiency on the brain. In this study, we fill this gap by showing for the first time that loss of A20 causes spontaneous cerebral inflammation, as demonstrated by robust microglial activation, reactive astrogliosis, endothelial activation, increased oxidative/nitrosative stress and expression of NFκB regulated pro-inflammatory soluble mediators such as IL-1β, TNF, IL-6 and MCP-1 in the brain.

By immunostaining, using F4/80 as microglia cell surface marker, we noted the presence of a significant number of hypertrophied microglia in A20 KO brain. This cell morphology, characterized by enlarged soma and thick cytoplasmatic projecting processes with few ramifications, is typical of microglia undergoing activation after CNS injury. This microglial phenotype contrasts with that of WT brain, which is ramified resting microglia, depicting radially long and thin projecting processes with fine ramifications. We confirmed microglial activation by demonstrating heightened A1 mRNA expression in the brains of A20 KO mice, as compared to WT. Expression of A1, a *BCL2* gene family member, in the CNS is restricted to microglia, and is uniquely upregulated when these cells undergo activation [38,39].

A20 KO brain also display enhanced astrogliosis, as morphologically evidenced by hypertrophy of astrocyte cell body and glial processes, together with increased expression of the intermediate filament GFAP, an early and sensitive biomarker of astrogliosis [62].

Furthermore, we documented *in vitro* that A20 deficient astrocytes and microglia are hyper reactive to inflammatory stimuli. Astrocytes isolated from A20 KO brain and A20-silenced primary mouse astrocytes and N13 microglia cells produced significantly higher amounts of IL-6 in response to inflammatory stimuli than WT and control cells. This accords with A20 being a negative feedback regulator of inducible NFκB-dependent genes, such as IL-6 [63], and corroborates work by Wang *et al*. showing enhanced TNF-mediated IκBα phosphorylation/NFκB activation in A20 KO astrocytes [25]. IL-6 plays a dual role in the CNS. IL-6 KO mice that suffer compromised inflammatory responses, increased oxidative stress, impaired neuroglial activation and decreased lymphocyte recruitment, show a slower rate of recovery and healing in several models of neuroinflammatory, degenerative and traumatic brain injury [64]. On the other hand, excessively high intracerebral IL-6 levels aggravate brain injury and damage by causing abnormal immune activation, decreased neurogenesis and differentiation of neural stem/progenitor cells into neurons [64].

A20 deficient astrocytes and microglia also produced significantly higher TNF and consequently higher IL-6 levels following engagement of the Toll-like receptor (TLR) signaling by LPS treatment. Altogether, our *in vivo* and *in vitro* data ascertain the critical role of A20 in regulating glial activation. In that regard, A20 deficient glia display a similar hyper-reactive pattern to A20 KO peritoneal macrophages, that is sustained IκBα degradation and higher TNF production in response to thioglycollate or LPS treatment [65]. As a result of microglial activation and reactive astrogliosis, A20 deficient brains bathe in a heightened pro-inflammatory milieu, as evidenced by significantly higher expression of TNF, IL-1β, IL-6 and MCP-1 in CX and HC of A20 KO mice as compared to WT.

Upregulation of the NADPH oxidase subunit gp91^phox^ and of the high throughput NOS, iNOS, is a distinctive hallmark of glial activation [66]. Gp91^phox^ deficient mice do not mount a robust ROS response following traumatic brain injury, and hence are relatively protected from cerebral damage [67]. iNOS, on the other hand, seems to have a dichotomous role in the brain. While absence of iNOS could impair neurogenesis after stroke, suggesting importance for CNS repair [68], excessive expression of iNOS is generally deleterious, and accordingly, genetic or pharmacologic knockdown of iNOS reduces tissue damage and neuronal death in animal models of brain injury [69,70]. Our data demonstrate that both gp91^phox^ and iNOS expression are increased at baseline in the brain of A20 KO mice. This would result in heightened local production of NO and of O^2-^, generating highly toxic peroxynitrite radicals that promote protein nitration, particularly damaging to the CNS [71]. Indeed, we observed increased nitrotyrosine immunostaining in A20 KO when compared to WT brains. Upregulation of gp91^phox^ and iNOS gene expression in reactive astrocytes and microglia is NFκB-dependent [72,73], explaining their enhanced cerebral levels in the absence of A20. Importantly, NFκB activation is also a downstream target of NADPH oxidase products [72], hence the self-feeding inflammatory and pro-oxidant spiral observed in A20 KO brain. In addition, our data also suggest that A20 KO brains are unable to mount an appropriate antioxidant response, as they fail to significantly upregulate the expression of antioxidant genes such as HO-1 despite Nrf2 upregulation, which further amplifies oxidative stress, possibly causing heightened axonal damage, as suggested by decreased immunostaining for neurofilaments in KO brains.

Activation and loss of brain EC is another feature of inflammation driven CNS injury [74]. Maintenance of endothelial homeostasis and of the unique phenotype of the BBB depends on tight interaction between EC, perivascular glial cells and neurons via direct cell-cell contact or through soluble factors to maintain the BBB. Having demonstrated that A20 KO mice suffer important gliosis, we checked the status of brain EC in these mice. As anticipated, astrogliosis and microglial activation corresponded with overt EC activation in A20 KO brain vasculature, as demonstrated by increased expression of the adhesion molecules VCAM-1, ICAM-1 and endothelial specific E-selectin, as well as the chemokine MCP-1, although the latter may be a product of glial cells, in addition to EC [75]. Increased endothelial activation in brains of A20 KO mice is in keeping with the well-documented anti-inflammatory and homeostatic function of A20 in EC [15,76], and agrees with our recent data demonstrating that mere partial loss of A20 aggravates the inflammatory phenotype of the endothelium in a vascular allograft model of transplant arteriosclerosis (Lee *et al*., manuscript in preparation).

Notably, A20 KO mice analyzed in this work were not exposed to exogenous toxic substances, pathogens or surgical procedure, raising questions regarding the primary signals/mediators triggering spontaneous neuroinflammation in these mice.

Data demonstrating that spontaneous multi-organ inflammation observed in A20 KO mice resolves when the TLR adapter MyD88 is simultaneously knocked out (A20/MyD88 double knockout), implicate pathogen-associated molecular patterns from commensal bacteria in driving the inflammatory process [77]. We hypothesize that similar mechanisms might drive spontaneous neuroinflammation in these mice. Indeed, the BBB may be breached in A20 KO and as a result, greater levels of LPS may cross the BBB and directly activate TLR expressing microglia. Alternatively, heightened EC inflammation and by consequence production of cytokines, [78,79] would engage NFκB signaling and activate microglia [80]. Activated microglia in turn would cause reactive astrogliosis [81], creating a paracrine and autocrine feedback loop whereby microglia- and astrocyte-derived factors would regulate each other, promoting a self-sustained pro-inflammatory environment. We favor the latter scenario as we failed to show any significant disruption of the BBB in A20 KO mice, at least at baseline.

In contrast to our observations in whole-body A20 KO mice, astrocyte, neuronal and neuroectodermal (astrocytes, neurons and oligodendrocytes) specific A20 KO do not cause spontaneous inflammation in the CNS [25]. This suggests that A20 knockdown on microglia and/or brain EC is required to cause spontaneous inflammation of the CNS, which would agree with our hypothesis placing these two cell types at the initiation of the neuroinflammatory process. Whether specific A20 KO in any or both of these cells is sufficient to cause neuroinflammation, or whether A20 KO in all brain cells (microglia, astrocytes, neurons, oligodendrocytes, endothelial cells) is required to have the phenotype we observe remains to be determined.

Limited survival of A20 KO animals restricts our study in terms of gauging their responses in several animal models of cerebral diseases. On the other hand, our laboratory has evidence that A20 HT mice, that do not present any apparent signs of pathology, uncover a significant phenotype upon challenge. In particular, we have strong indication that partial hepatectomy, a benign procedure in WT mice, harbors high lethality in A20 HT mice (Studer *et al*., manuscript submitted). High lethality in these mice stems from inadequate liver regeneration that partly results from heightened inflammation. Accordingly, we set up to check the baseline brain phenotype of A20 HT mice. Interestingly, our findings show that partial loss of A20 results in mild cerebral inflammation, as demonstrated by a moderate yet consistent increase in pro-inflammatory and oxidative/nitrosative stress markers in the CX and HC of A20 HT mice. Those findings are highly significant, given recently described SNPs in the A20/*TNFAIP3* locus, imparting decreased A20 expression or function (NFκB inhibition), that were linked with auto-immune and pro-inflammatory pathologies such as systemic lupus erythematosus, rheumatoid arthritis and multiple sclerosis [82,83]. These ‘risky’ *TNFAIP3* SNPs, akin to those seen in A20 HT mice, may cause low-grade inflammation in the brain, predisposing patients to neuroinflammation and neurodegenerative diseases.

Neuroinflammation in brains of A20 HT mice is bound to increase with aging, and possibly metabolic diseases such as diabetes. It is well documented that microglial cells in aging brains, including those of mice, demonstrate a sensitized phenotype, that is, they release higher amounts of pro-inflammatory mediators upon activation [84]. In addition, our group has shown that A20 protein levels decrease in the context of diabetes, as a result of increased proteasomal degradation stemming from high glucose driven post-translational modifications, namely o-glycosylation and ubiquitination [85].

## Conclusion

Altogether, our data uncover the cerebral phenotype of A20 deficient mice that suffer spontaneous neuroinflammation as depicted by heightened gliosis and endothelial cell activation, feeding into a spiral of local cytokine and chemokine production together with increased oxidative/nitrosative stress, all of which culminate in neuronal damage. Future studies using A20 HT as a model for chronic spontaneous low-grade neuroinflammation may help clarify the role of A20 in brain inflammation related to aging or metabolic diseases, as well as inflammatory neurodegenerative diseases such as PD, AD, stroke or trauma.

## Abbreviations

AD: Alzheimer’s disease; ANOVA: analysis of variance; BBB: blood brain barrier; CCL-2: chemokine (C-C motif) ligand 2; CNS: central nervous system; CX: cerebral cortex; DAB: 3,3’-diaminobenzidine tetrahydrochloride; DAPI: 4′,6-diamidino-2-phenylindole; DMEM: Dulbecco’s modified Eagle medium; EC: endothelial cells; ECL: enhanced chemiluminescence; ELISA: enzyme linked immunosorbent assay; eNOS: endothelial nitric oxide synthase; FBS: fetal bovine serum; GAPDH: glyceraldehyde 3-phosphate dehydrogenase; GFAP: glial fibrillary acidic protein; HC: hippocampus; HO-1: heme oxigenase-1; HRP: horseradish peroxidase; HT: heterozygous; ICAM-1: intercellular adhesion molecule-1; IF: immunofluorescence; IHC: immunohistochemistry; IL-1β: interleukin-1 beta; IL-6: interleukin-6; iNOS: inducible nitric oxide synthase; KO: knockout; LPS: lipopolysaccharide; MCP-1: monocyte chemoattractant protein 1; MS: multiple sclerosis; NF: neurofilament; NFκB: nuclear factor kappa B; NTY: nitrotyrosine; nNOS: neuronal nitric oxide synthase; NO: nitric oxide; Nrf2: nuclear factor nuclear factor erythroid 2 related factor 2; NSAIDs: non-steroidal anti-inflammatory drugs; PD: Parkinson’s disease; PVDF: polyvinylidene fluoride; qPCR: quantitative reverse transcriptase-polymerase chain reaction; S100β: S100 calcium-binding protein beta; SDS-PAGE: sodium dodecyl sulphate-polyacrylamide gel electrophoresis; siRNA: silence RNA; SNPs: single nucleotide polymorphisms; SYBR: Sybergreen; TLR: Toll-like receptor; TNF: tumor necrosis factor alpha; TNFAIP3: tumor necrosis factor alpha-induced protein 3; VCAM-1: vascular cell adhesion protein-1; WT: wild type.

## Competing interests

The authors declare that they have no competing interests.

## Authors’ contribution

RPG and CGS designed and performed experiments, participated in the critical analysis and interpretation of the data, performed statistical analysis of the data and wrote the manuscript. EC carried out the immunoassays (IHC). HPM carried out silence RNA experiments. CF participated in the critical analysis and interpretation of the results, contributed reagents/materials tools and helped write the manuscript. AM generated and provided the A20 knockout mice. All authors read and approved the final manuscript.

## Supplementary Material

Additional file 1: Figure S1 Absence of non-specific staining in negative controls for immunohistochemistry. Primary antibodies were omitted and immunohistochemistry was performed using secondary IgG anti-goat, anti-rabbit, anti-rat and anti-hamster in cerebral cortex (CX) and hippocampus (HC). Bar = 50 μm, magnification = 200x. Click here for file

Additional file 2: Figure S2 Transfection of mouse primary astrocytes and microglia cell line N13 with A20 silencing RNA significantly reduces LPS-induced upregulation of A20 mRNA. A20 mRNA levels measured by qPCR in **A**. mouse primary astrocytes and **B**. microglia cell line N13 1 hour after LPS (1μg/mL) stimulation. Graphs represent relative mRNA levels after normalization by βactin. NS: non-stimulated cells. Ctrl: non-transfected control cells. A20 siRNA: cells transfected with A20 silence RNA. C siRNA: cells transfected with All Star control silence RNA. *P < 0.05, **P < 0.01 and ***P < 0.001. Click here for file

Additional file 3: Figure S3 HO-1 levels are unchanged in cerebral cortex and hippocampus of A20 deficient mice. **A**. HO-1 mRNA levels in cerebral cortex (CX) and hippocampus (HC) of wild type (WT), A20 heterozygous (HT) and A20 knockout (KO) mice, measured by qPCR. Graph shows of relative RNA levels after normalization with βactin. Results are expressed as mean ± SEM for six to seven animals per genotype. Click here for file

Additional file 4: Figure S4 Loss of A20 does not induce spontaneous changes in blood brain barrier (BBB) permeability. **A**. Evan’s blue dye (EB) extravasation: Wild type (WT), A20 heterozygous (HT) and A20 knockout (KO) mice using were intravenously injected with 2% EB solution. 1.5 hours after injection, animals were transcardially perfused with saline and brains were processed to measure fluorescence. Graph shows optical density (OD) at 620 nm. **B**. S100β protein levels in serum from WT, HT and KO mice, measured by ELISA. Results are expressed as mean ± SEM for three to six animals per genotype. Click here for file

Additional file 5 Supplementary Experimental Procedures. Click here for file
