# Supplementary figures and images for: A20 deficiency causes spontaneous neuroinflammation in mice
Source: J Neuroinflammation. 2014 Jul 16;11:122. doi: 10.1186/1742-2094-11-122 (PMC4128606; doi:10.1186/1742-2094-11-122)

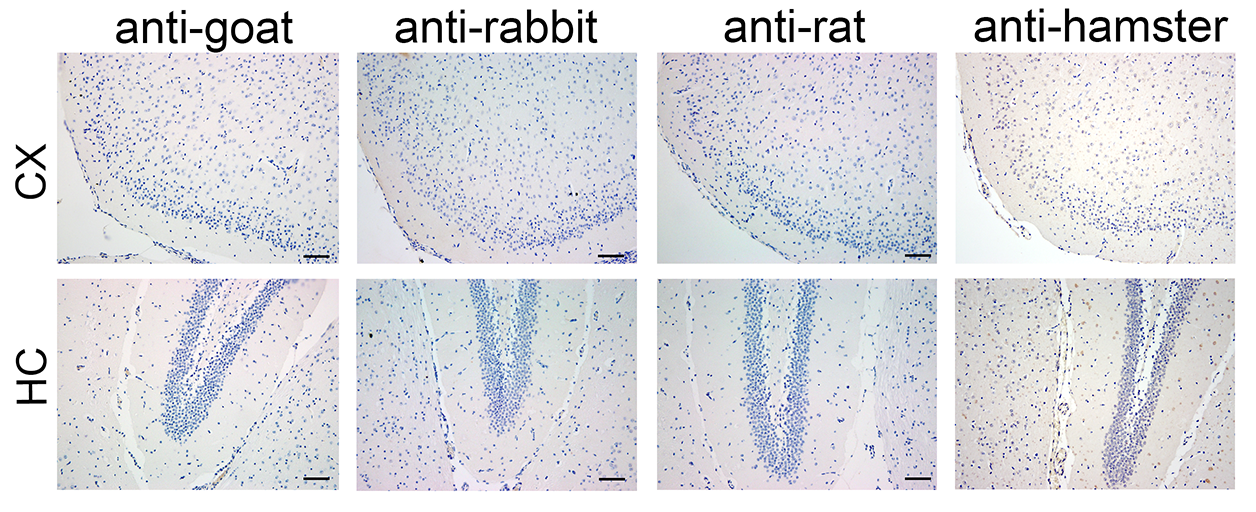

Supplement: Additional file 1: Figure S1 — Absence of non-specific staining in negative controls for immunohistochemistry. Primary antibodies were omitted and immunohistochemistry was performed using secondary IgG anti-goat, anti-rabbit, anti-rat and anti-hamster in cerebral cortex (CX) and hippocampus (HC). Bar = 50 μm, magnification = 200x. [file 1742-2094-11-122-S1.tiff]

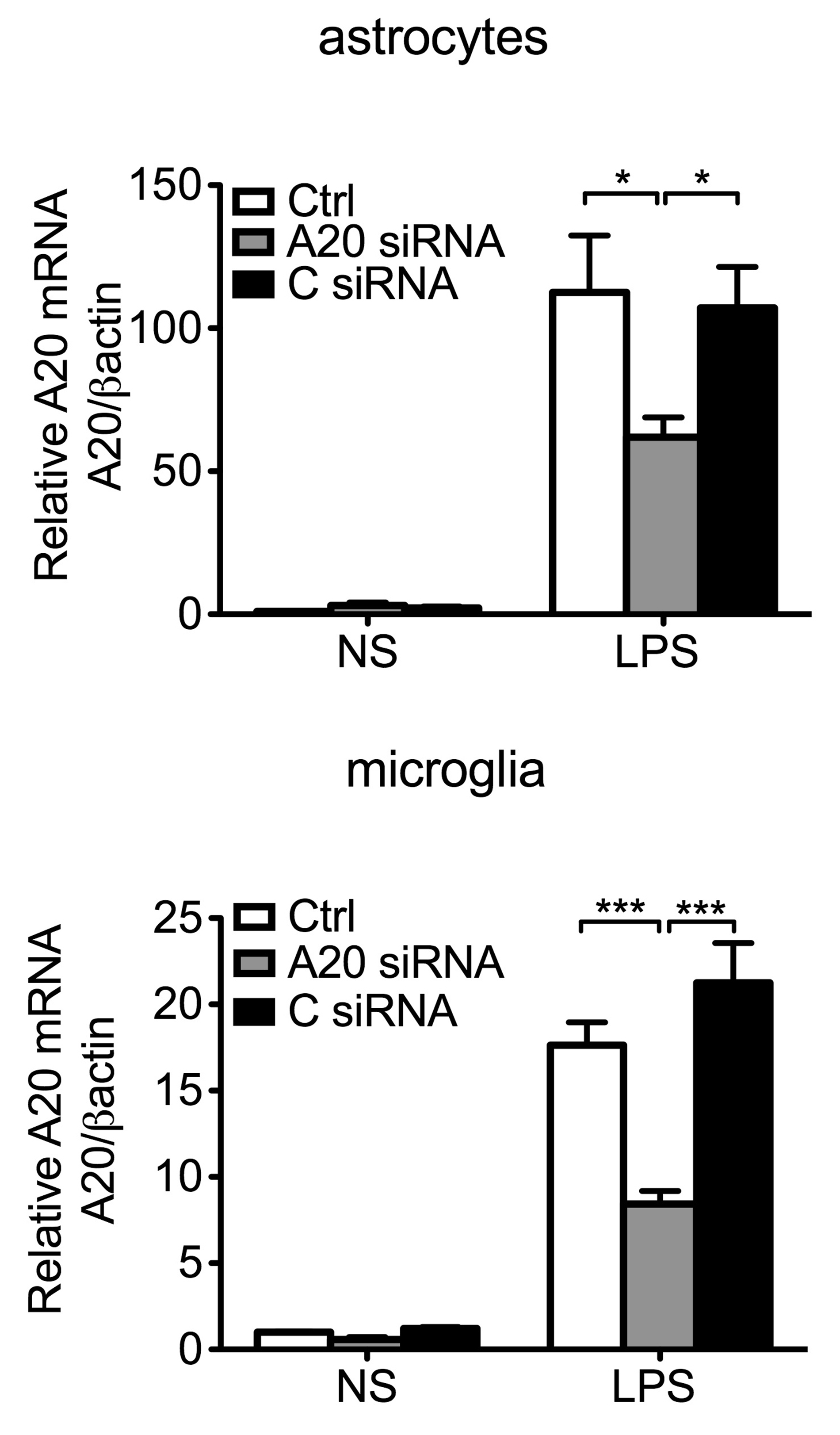

Supplement: Additional file 2: Figure S2 — Transfection of mouse primary astrocytes and microglia cell line N13 with A20 silencing RNA significantly reduces LPS-induced upregulation of A20 mRNA. A20 mRNA levels measured by qPCR in A. mouse primary astrocytes and B. microglia cell line N13 1 hour after LPS (1μg/mL) stimulation. Graphs represent relative mRNA levels after normalization by βactin. NS: non-stimulated cells. Ctrl: non-transfected control cells. A20 siRNA: cells transfected with A20 silence RNA. C siRNA: cells transfected with All Star control silence RNA. *P < 0.05, **P < 0.01 and ***P < 0.001. [file 1742-2094-11-122-S2.tiff]

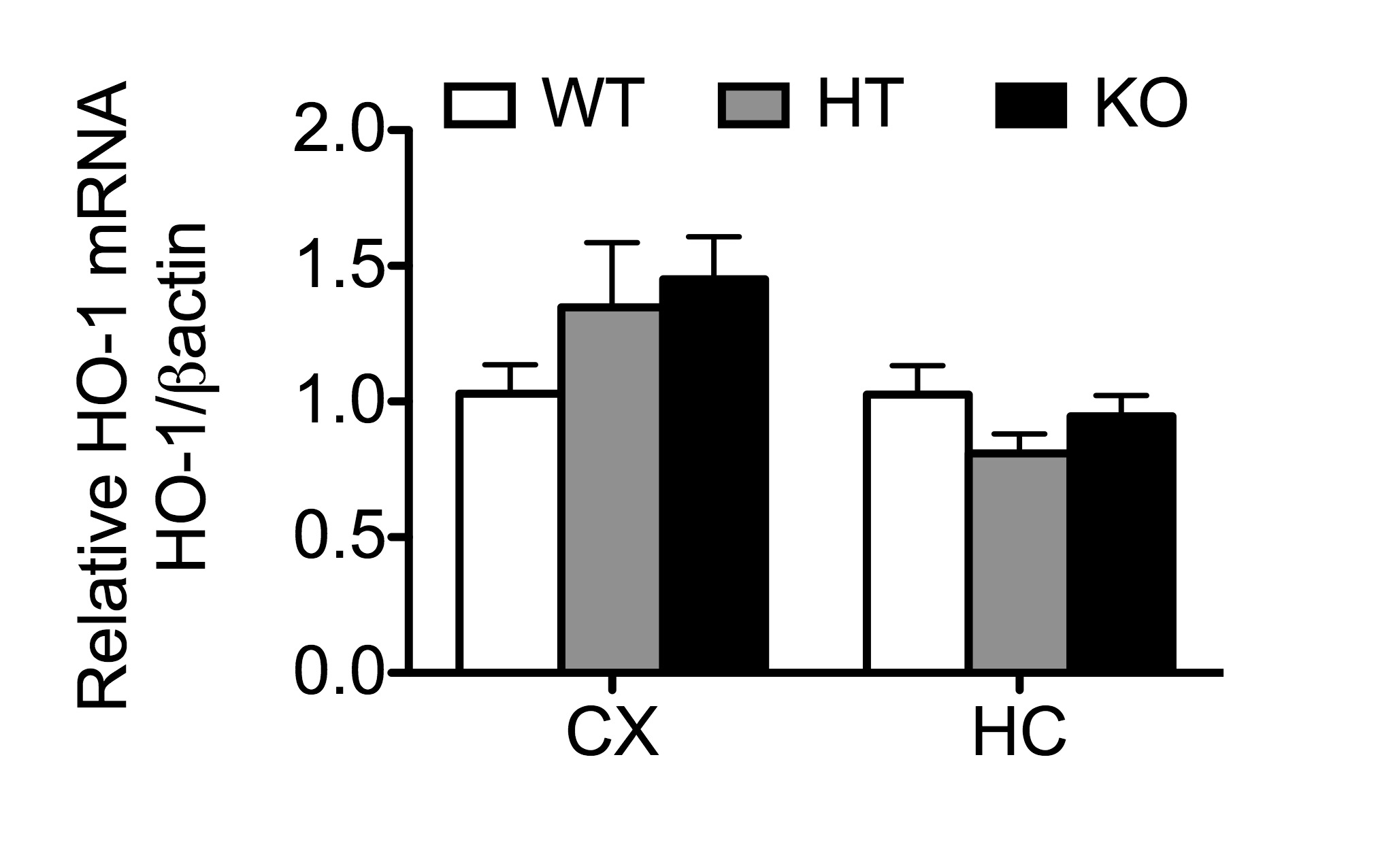

Supplement: Additional file 3: Figure S3 — HO-1 levels are unchanged in cerebral cortex and hippocampus of A20 deficient mice. A. HO-1 mRNA levels in cerebral cortex (CX) and hippocampus (HC) of wild type (WT), A20 heterozygous (HT) and A20 knockout (KO) mice, measured by qPCR. Graph shows of relative RNA levels after normalization with βactin. Results are expressed as mean ± SEM for six to seven animals per genotype. [file 1742-2094-11-122-S3.tiff]

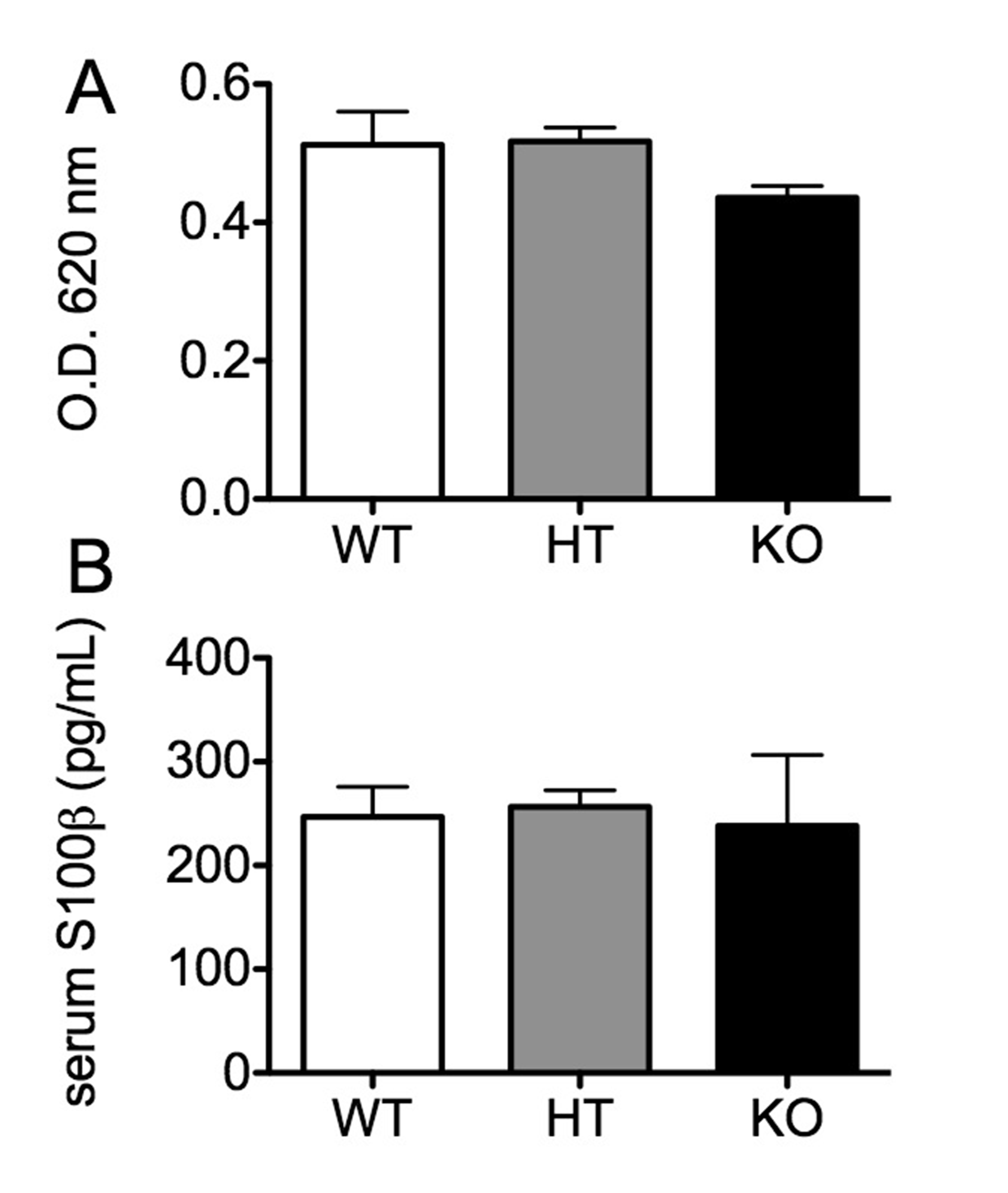

Supplement: Additional file 4: Figure S4 — Loss of A20 does not induce spontaneous changes in blood brain barrier (BBB) permeability. A. Evan’s blue dye (EB) extravasation: Wild type (WT), A20 heterozygous (HT) and A20 knockout (KO) mice using were intravenously injected with 2% EB solution. 1.5 hours after injection, animals were transcardially perfused with saline and brains were processed to measure fluorescence. Graph shows optical density (OD) at 620 nm. B. S100β protein levels in serum from WT, HT and KO mice, measured by ELISA. Results are expressed as mean ± SEM for three to six animals per genotype. [file 1742-2094-11-122-S4.tiff]
