# Supplementary material for: A20 deficiency causes spontaneous neuroinflammation in mice
Source: J Neuroinflammation. 2014 Jul 16;11:122. doi: 10.1186/1742-2094-11-122 (PMC4128606; doi:10.1186/1742-2094-11-122)
Supplement: Additional file 5 — Supplementary Experimental Procedures. [file 1742-2094-11-122-S5.docx]

**Additional file 5: Experimental Procedures:**

**Quantitative Evaluation of Evan’s blue dye (EB) extravasation:** Vascular permeability was quantitatively evaluated by fluorescent detection of extravasated EB. Briefly, 2% Evans blue dye in saline was injected intravenously (4 ml/kg of body weight). 90 minutes after injection, mice were deeply anesthetized with transcardially perfused with saline until colorless perfusion fluid was obtained from the right atrium. After decapitation, the brains were weighted and homogenized in 10-fold volume of N,N-dimethylformamide and incubated overnight at 55°C. Samples were centrifuged and optical density (O.D.) of the supernatant at 620nm was determined by a microplate reader.

**Circulating S100β levels:** S100β levels were measured in serum samples of 4 to 5-week-old mice using mouse S100β (S100 calcium binding protein β) ELISA kit from Bioaspect Inc (Toronto, ON, Canada), according to manufacture’s instructions.
